# Experimental Horizontal Gene Transfer of Methylamine Dehydrogenase Mimics Prevalent Exchange in Nature and Overcomes the Methylamine Growth Constraints Posed by the Sub-Optimal *N*-Methylglutamate Pathway

**DOI:** 10.3390/microorganisms3010060

**Published:** 2015-03-10

**Authors:** Dipti D. Nayak, Christopher J. Marx

**Affiliations:** 1 Organismic and Evolutionary Biology, Harvard University, Cambridge, MA 02138, USA; E-Mail: dipti.nayak@gmail.com; 2 Biological Sciences, University of Idaho, Moscow, ID 83844, USA; 3 Faculty of Arts and Sciences Center for Systems Biology, Harvard University, Cambridge, MA 02138, USA; 4 Institute for Bioinformatics and Evolutionary Studies, University of Idaho, Moscow, ID 83844, USA

**Keywords:** Methylotrophy, Methylamine, Experimental Evolution, *Methylobacterium*, Horizontal Gene Transfer (HGT)

## Abstract

Methylamine plays an important role in the global carbon and nitrogen budget; microorganisms that grow on reduced single carbon compounds, methylotrophs, serve as a major biological sink for methylamine in aerobic environments. Two non-orthologous, functionally degenerate routes for methylamine oxidation have been studied in methylotrophic Proteobacteria: Methylamine dehydrogenase and the *N-*methylglutamate pathway. Recent work suggests the *N*-methylglutamate (NMG) pathway may be more common in nature than the well-studied methylamine dehydrogenase (MaDH, encoded by the *mau* gene cluster). However, the distribution of these pathways across methylotrophs has never been analyzed. Furthermore, even though horizontal gene transfer (HGT) is commonly invoked as a means to transfer these pathways between strains, the physiological barriers to doing so have not been investigated. We found that the NMG pathway is both more abundant and more universally distributed across methylotrophic Proteobacteria compared to MaDH, which displays a patchy distribution and has clearly been transmitted by HGT even amongst very closely related strains. This trend was especially prominent in well-characterized strains of the *Methylobacterium extroquens* species, which also display significant phenotypic variability during methylamine growth. Strains like *Methylobacterium extorquens* PA1 that only encode the NMG pathway grew on methylamine at least five-fold slower than strains like *Methylobacterium extorquens* AM1 that also possess the *mau* gene cluster. By mimicking a HGT event through the introduction of the *M. extorquens* AM1 *mau* gene cluster into the PA1 genome, the resulting strain instantaneously achieved a 4.5-fold increase in growth rate on methylamine and a 11-fold increase in fitness on methylamine, which even surpassed the fitness of *M. extorquens* AM1. In contrast, when three replicate populations of wild type *M. extorquens* PA1 were evolved on methylamine as the sole carbon and energy source for 150 generations neither fitness nor growth rate improved. These results suggest that the NMG pathway permits slow growth on methylamine and is widely distributed in methylotrophs; however, rapid growth on methylamine can be achieved quite readily through acquisition of the *mau* cluster by HGT.

## 1. Introduction

**Figure 1:**
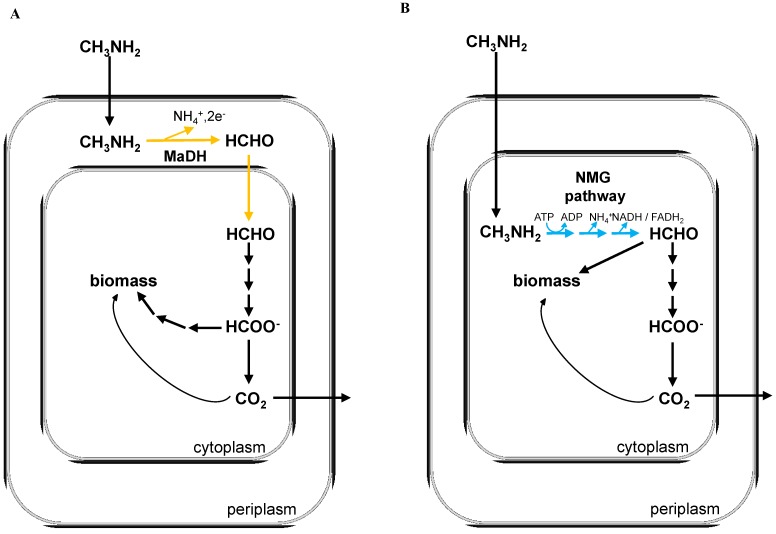
(**A**) A schematic of methylamine metabolism using the methylamine dehydrogenase (encoded by the *mau* gene cluster) for methylamine oxidation (orange). During methylamine growth mediated by methylamine dehydrogenase, formate is the branch point of C_1_ metabolism and the tetrahydrofolate (H_4_F) dependent pathway is essential for C_1_ assimilation. (**B**) A schematic of methylamine metabolism using the *N*-methylglutamate pathway for methylamine oxidation (blue). During methylamine growth mediated by the *N*-methylglutamate (NMG) pathway, formaldehyde is the branch point of C_1_ metabolism and the H_4_F dependent pathway is not used at all for C_1_ assimilation.

The simplest methylated amine, mono-methylamine (CH_3_NH_2_; MA) is a toxic, inflammable organic compound that plays an important role in the carbon and nitrogen biogeochemical cycles [1,2,3,4,5,6,7] and contributes significantly to the biogenesis of greenhouse gases like methane [8,9]. MA is produced during the decarboxylation of organic matter [5,6], anaerobic degradation of proteins and osmolytes [10] and also as a by-product of several industries like fish processing [6] and pesticide production [6]. In aerobic environments, methylotrophic bacteria that grow on reduced single carbon (C_1_) compounds like methane and methanol [11,12,13] are one of the major sinks for MA [2,4,7]. Two functionally degenerate yet non-orthologous routes for MA oxidation have been characterized in methylotrophic Proteobacteria. One route is mediated by a single enzyme called methylamine dehydrogenase (MaDH) [11,13,14], whereas the alternate route is mediated by three distinct enzymes of the *N-*methylglutamate (NMG) pathway [15,16] (Figure 1A,B). The NMG pathway is also observed in various taxa of non-methylotrophic proteobacteria where it has been shown to facilitate the utilization of MA as a nitrogen source [7].

More than two decades ago, cultivation and enrichment based studies of MA growth in methylotrophs honed in on the genetic and biochemical characterization of MaDH, an enzyme with a unique, chemically modified amino acid coenzyme [17,18,19] that was found in model organisms like *Paracoccus* *denitrificans* Pd122 [20] and *Methylobacterium extorquens* AM1 (referred to as AM1 here on) [14,17]. In AM1, MaDH and ancillary proteins (required for protein maturation and electron transport) are encoded by the *mau* gene cluster [14], which is present in a 10 kb genomic region that is flanked by two insertion sequence (IS) elements of the ISMex15 family [21]. MaDH is a periplasmic enzyme that oxidizes MA in a single-step to produce free formaldehyde and ammonia, with the resulting electrons being passed to the electron transport chain [11,18,19] (Figure 1A). Similar to its fate during methanol growth in AM1, free formaldehyde produced by MaDH is oxidized to formate by a series of tetrahydromethanopterin (H_4_MPT) dependent reactions in the cytoplasm [22]. Subsequently, a fraction of the formate generated is further oxidized to CO_2_ by a panel of formate dehydrogenases [23,24] and the rest gets assimilated into various components of biomass via a tetrahydrofolate dependent (H_4_F) pathway [13,25], the serine cycle [11,26,27], and the ethyl-malonyl CoA pathway [28].

In contrast to previous findings, recent studies with *Methyloversatilis universalis* FAM5 [15], *Methylobacterium extorquens* PA1 (referred to as PA1 from here on) (16) and various other bacteria [29,30] have shown that these strains can grow on MA despite lacking the *mau* gene cluster. In these methylotrophs, MA oxidation is mediated by the NMG pathway that, unlike MaDH, requires three enzymatic steps and generates two amino acid derivatives as metabolic intermediates [31,32,33,34] (Supplementary Figure S1). Furthermore, the NMG pathway is cytoplasmic, requires an ATP, and generates either FADH_2_ or NADH [15,32,33] (Figure 1B). Finally, in PA1, it also has been shown that MA growth mediated by the NMG pathway reroutes C_1_ flux such that the H_4_F dependent C_1_ transfer pathway is no longer used for C_1_ assimilation [16]. Thus, beyond possible kinetic differences, distinct localization and cofactor coupling are also likely to influence the efficiency and yields of these two functionally degenerate MA oxidation pathways as well.

Of late, metagenomic data is revealing that the *mau* gene cluster is significantly less abundant than genes of the NMG pathway in freshwater [7,35] as well as saline environments [4,36]. While the biochemistry and genetics of each functionally degenerate route for MA oxidation is well established at this point [11,13,14,15,16,17], to our knowledge no study has contrasted MA oxidation mediated by the MaDH route *versus* the NMG pathway to understand the physiological or adaptive constraints that govern the distribution of these MA oxidation modules in the environment. Based on the evidence that the *mau* gene cluster in AM1 is surrounded by IS elements, HGT has been speculated as a means of transmission of MaDH [21]. However, the evolutionary paradigm controlling the distribution of these two MA oxidation routes in extant methylotrophs has never been analyzed. Nor is it known whether the presence of a specific MA oxidation module influences growth in a predictable and consistent way. Well-studied strains of the *Methylobacterium extorquens* species serve as a tractable system in which to compare these two functionally degenerate routes for MA oxidation. Even though *M. extorquens* species are more than 99% identical at the 16s rRNA locus [37] and share a core methylotrophy-specific metabolic repertoire [38], stark genotypic differences have been observed on MA [16,38]. Whereas all *M. extorquens* strains possess the NMG pathway [21,37,38], AM1 and CM4 also contain MaDH [21] (Figure 1A,B). Furthermore, these genotypic differences are also known to influence MA growth: AM1 can grow on MA with rates that are five-fold higher than those observed for PA1 [16,38].

In this study, we used two complementary yet distinct approaches to contrast the evolution and physiology of these two alternate MA oxidation pathways in methylotrophs. First, examining the phylogeny and synteny of each pathway, we uncovered the patterns of evolutionary history of the NMG pathway and MaDH in sequenced methylotrophic Proteobacteria. While the NMG pathway is more abundant and universally distributed, especially in methylotrophic genera of the Alpha- and Beta-proteobacteria, the phylogeny of genes encoding MaDH was indicative of recent HGT within and across distant clades of the Proteobacteria. Next, we zoomed in and contrasted the role of evolution by mutation and natural selection *versus* evolution by HGT during MA growth in a well-characterized strain of the *M. extroquens* species. We sought to test in PA1 the relative ease of obtaining rapid growth on MA via acquisition of MaDH via HGT, *versus* by evolving improved function of the NMG pathway. By simply introducing the *mau* gene cluster on a plasmid to simulate the HGT events inferred to have occurred naturally in strains AM1 and CM4 [21], the MA fitness of the PA1 transconjugant was even higher than AM1. In contrast, even after 150 generations of laboratory evolution on MA as the sole carbon and energy source, neither competitive fitness nor growth rate improved. These results provide a counter example to the typical pattern in metabolic engineering of poor growth immediately after introduction of a foreign metabolic module [39,40], as well as the common notion in microbial evolution that strains with lower fitness tend to adapt more rapidly [41,42]. Furthermore, we directly demonstrate that an HGT event can instantaneously overcome physiological constraints imposed by certain metabolic pathways and dramatically change the performance and fitness of an organism on a growth substrate.

## 2. Experimental Section

### 2.1. Chemicals and Media

All chemicals were purchased from Sigma-Aldrich (St. Louis, MO, USA) unless otherwise noted. *Escherichia coli* were grown in Luria Bertani broth at 37 °C with the standard antibiotic concentrations. Standard growth conditions for *M. extroquens* PA1 and *M. extorquens* AM1 utilized a modified version of Hypho minimal medium consisting of: 100 mL phosphate salts solution (25.3 g of K_2_HPO_4_ plus 22.5 g Na_2_HPO_4_ in 1 L deionized water), 100 mL sulfate salts solution (5 g of (NH_4_)_2_SO_4_ and 2 g of MgSO_4_·7 H_2_O in 1 L deionized water), 799 mL of deionized water, and 1 mL of trace metal solution [43]. Filter-sterilized carbon sources were added just prior to inoculation in liquid minimal media with a final concentration of 3.5 mM for sodium succinate and 20 mM for methylamine hydrochloride.

### 2.2. Phylogenetic Analysis

Amino acid similarity to *mauA* and *mauB* (which encode the small and large subunit of MaDH) from *M. extorquens* AM1 [14] and *gmaS, mgsC* and *mgdC* (which encode γ-glutamylmethylamide synthetase, and subunits of *N-*methylglutamate synthase and *N-*methylglutamate dehydrogenase respectively) from *M. extorquens* PA1 [16] were used to detect the presence of MaDH and/or the *N-*methylglutamate pathway, respectively, in sequenced methylotrophic Proteobacterial genomes deposited in NCBI. The criterion for detecting genes representing each of these MA oxidation pathways was based upon a sequence similarity and sequence coverage cutoff. The cutoff was determined based on either: (a) parameter values below which a significant, sharp rise in the e-value of the BLAST search results was observed (*mauA* and *mauB*) or (b) parameter values below which functionally distinct homologs were observed (*gmaS*, *mgsC*, *mgdC*). Sequence coverage of >96% and >85%, sequence identity of >54% and >43% (corresponding to an e-value of 4e-55 and 2e-51) was used as the criterion for detecting *mauA* and *mauB* like sequences, respectively, in the genomes of sequenced methylotrophs within the Proteobacteria. Sequence coverage of >96%, >96% and >87% and sequence identity of >40%, >81%, and >35% was used as the threshold for the detection of *gmaS, mgsC* and *mgdC* like sequences in the genomes of sequenced methylotrophs within the Proteobacteria. Sequence alignment was conducted using the MUSCLE alignment software [44] with a maximum of 127 iterations. ML (Maximum Likelihood) phylogenetic analysis was performed with the PhyML package built in Geneious version 5.4 [45] using the HKY85 substitution model [46] and the Dayhoff substitution model [47] for nucleotide and amino acid sequences, respectively, and employed 500 bootstrap resamplings. The proportion of invariable sites, gamma distribution parameters, and transition/transversion ratios (only for nucleotide sequences) were estimated; four substitution rate categories were used, and the topology, branch length, as well as rate parameters were optimized.

### 2.3. Experimental Evolution

Three independent colonies of the ∆*cel* strain of *M. extorquens* PA1 lacking cellulose biosynthesis (CM2730) (Table 1) [48] were isolated from Hypho, succinate, agar (2% w/v) plates and were inoculated in 50 mL flasks with 10 mL of Hypho minimal media with 20 mM methylamine hydrochloride to initiate three replicate populations for experimental evolution. Flasks were incubated in a 30 °C shaking incubator at 225 rpm for 3.5 days after which a 32-fold dilution (for the first 11 transfers) and a 128-fold dilution (for the next 14 transfers) of the culture was transferred into fresh media. At regular intervals, populations were frozen at −80 °C with 10% DMSO. *M. extorquens* AM1 contamination was tested after every transfer by using *mau* specific primers for PCR amplification from the population lysate. Evolved clones were obtained as independent colonies on 7.5 mM sodium succinate, 20 mM MA, Hypho agar (2% w/v) plates.

### 2.4. Fitness Assays

Competitive fitness was measured by competing strains against a fluorescently labelled ancestor (*M. extorquens* PA1 ∆*cel-∆hpt*::*P_tac_-mCherry* (CM3839) (Table 1) described elsewhere [49] using a modified version of a protocol described elsewhere [50,51]. Growth was initiated by transferring 10 μL freezer stock into 10 mL of Hypho medium with 3.5 mM succinate. Upon reaching stationary phase, a 32-fold dilution of the cultures was transferred into fresh medium with 20 mM MA. At the end of the acclimation phase, CM3839 and the test strain or the mixed population were mixed in equal proportions by volume and this initial mix (T_0_) was transferred 1:32 into fresh media with the same growth conditions as the acclimation phase. 450 μL of the T_0_ mix was mixed with 10% DMSO and frozen at −80 °C. A 500 μL sample at the end of the growth phase (T_1_) was collected and the ratio of CM3839 and the test strain or the mixed population before and after the growth phase was ascertained using flow cytometry. Cells were diluted appropriately such that at a flow rate of 0.5 μL/s on the LSRFortesssa (BD, Franklin Lakes, NJ, USA) ~1000 events/s would be recorded. Fluorescent mCherry was excited at 561 nm and measured at 620/10 nm. The competitive fitness was calculated as $W=\frac{\text{log}(\frac{R1*N}{R0})}{\text{log}(\frac{\left(1-R1\right)*N}{\left(1-R0\right)})}$ where *R*1 and *R*0 represent the population fraction of the test strain before and after mixed growth, and *N* represents the fold increase in the population density. Competitive fitness assays were conducted in triplicate unless specified. Data are reported as the mean fitness ± 95% confidence interval of the mean fitness value.

**Table 1:** *M. extorquens* strains and plasmids generated in this study.

Strains or Plasmid	Description	Reference
**Strains**		
CM4	*M. extorquens* CM4	[52]
DM4	*M. extorquens* DM4	[53]
BJ001	*M. extorquens* BJ001	[54]
CM2720	∆*cel M. extorquens* AM1	[55]
CM2730	∆*cel M. extorquens* PA1	[56]
CM3120	∆*katA*::*P_tac_-mCherry* in CM2720^a^	[49]
CM3839	∆*hpt*::*P_tac_-mCherry* in CM2730^b^	[49]
CM4408	E1	This study
CM4409	E2	This study
CM4410	E3	This study
**Plasmids**		
pAYC139	Plasmid (IncP, *tra,* Tet^R^) with *mau* gene cluster from *M. extorquens* AM1	[14]

*katA*: catalase; *hpt*: hypoxanthine phosphoribosyltransferase.

### 2.5. Growth Rate Measurement

The growth rate of *M. extorquens* strains containing the *mau* cluster was measured in 48-well microtiter plates (CoStar-3548, Corning Life Sciences, Tewksbury, MA, USA), shaken at 650 rpm in an incubation tower (Liconic USA LTX44 with custom fabricated cassettes, Woburn, MA, USA) that was maintained in a room at 30 °C and 80% humidity, using an automated platform as described elsewhere [38]. Since the growth rate of *M. extorquens* strains containing of the *N-*methylglutamate pathway was slower than the detection limit of the automated growth measurement platform, it was measured in 50 mL flasks that were incubated in an orbital, incubator with a shaking speed of 225 rpm that was maintained at 30 °C. Growth from freezer stocks at −80 °C was initiated by transferring 10 μL freezer stock into 10 mL of Hypho medium with 3.5 mM succinate. Upon reaching stationary phase (2 days), cultures were transferred 1:16 into 9.4 mL fresh medium with 20 mM MA and allowed to reach saturation in this acclimation phase (3.5 days), and diluted 1:32 again into 9.4 mL fresh medium with 20 mM MA for the measured (experimental) growth (3.5 days). A 50 μL aliquot of three replicate cultures, for each strain, was sampled every 8–10 h during the growth phase. Optical density of the culture was measured at 600 nm (OD_600_) using a spectrophotometer (Bio-Rad, Hercules, CA, USA). The dynamics and specific growth rate of cultures were calculated from the log-linear growth phase using CurveFitter [48,57]. Yield was measured as the maximum OD_600_ during the growth phase. Growth rate and yield reported for each strain and condition is the mean calculated from three biological replicates, unless otherwise noted.

## 3. Results

### 3.1. The N-Methylglutamate Pathway for Methylamine Oxidation is More Widely Distributed in Methylotrophic Proteobacteria than Methylamine Dehydrogenase

**Figure 2:**
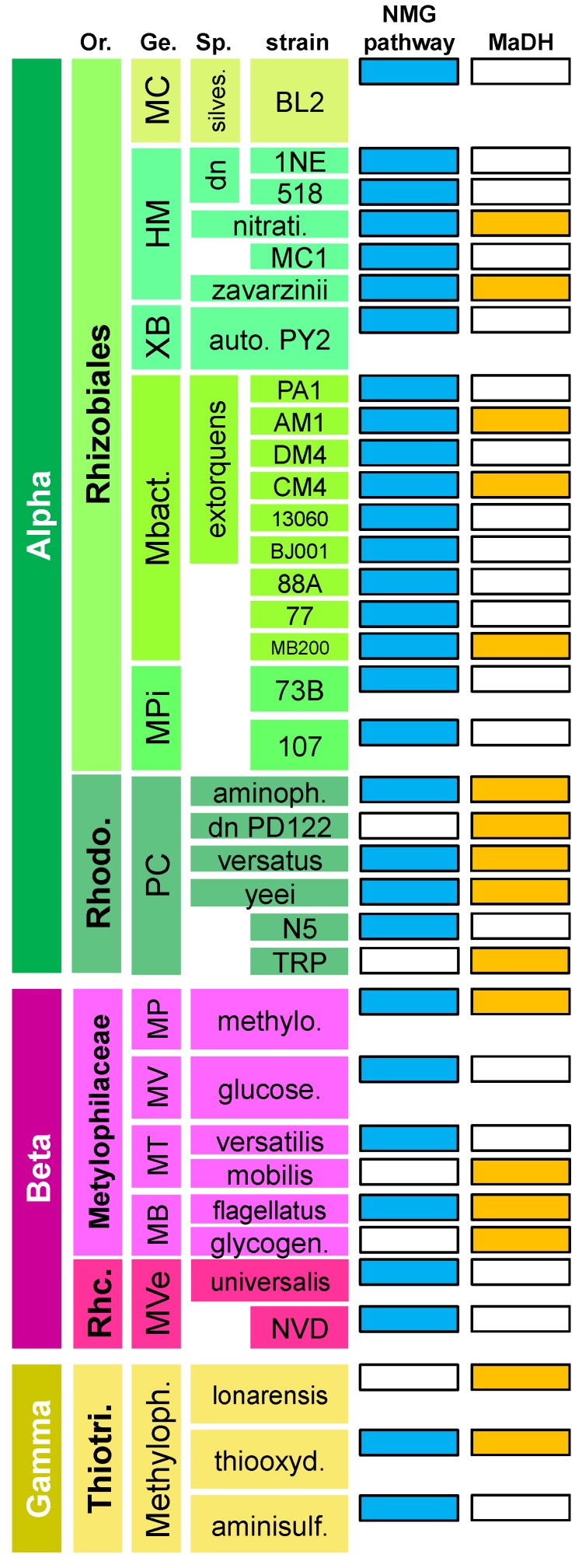
Distribution of genes of the *N-*methylglutamate pathway (in blue) and the *mau* gene cluster encoding methylamine dehydrogenase (in orange) in the genome of sequenced methylotrophs of the Alpha—(green), Beta—(pink), and Gamma—(yellow) Proteobacteria that contain at least one pathway for methylamine oxidation. Or.: Order; Ge.: Genus; Sp.: species; MC: *Methylocella*; silves.: *silvestris*; HM: *Hyphomicrobium*; dn: *denitrificans*; nitrati.: *nitrativorans*; XB: *Xanthobacter*; auto.: *autotrophicus*; Mbact.: *Methylobacterium*; Mpi: *Methylopila*; Rhodo: *Rhodobacterales*; PC: *Paracoccus*; aminoph.: *aminophilus*; MP: *Methylophilus*; methylo.: *methylotrophus*; MV: *Methylovorus*; glucose.: *glucosetrophus*; MT: *Methylotenera*; MB: *Methylobacillus*; glycogen.: *glycogenes*; Rhc.: *Rhodocyclales*; Mve: *Methyloversatilis*; Thiotri.: *Thiotricales*; Methyloph.: *Methylophaga*; thiooxyd.: *thiooxydans*; aminisulf.: *aminisulfidivorans*.

Sequence similarity to conserved genes of the NMG pathway and MaDH from *M. extorquens* species was used to determine the distribution for each of these MA oxidation pathways across sequenced methylotrophs within the Proteobacteria. The distribution of the NMG pathway and MaDH in methylotrophic Proteobacteria that contain either one or both routes for MA oxidation are shown in Figure 2. As indicated in Figure 2, the NMG pathway was found in approximately two times as many methylotrophic Proteobacteria compared to MaDH. The NMG pathway was particularly prevalent in members of the Rhizobiales family, where all of the 18 strains that possess genes for growth on MA have the NMG pathway and only 5 also encode the *mau* gene cluster. The NMG pathway was also more universally distributed; genes of this pathway were found in at least one sequenced member of all known methylotrophic genera in the Alpha- and Beta-proteobacteria except for the recently discovered methylotroph- *Methylibium petroleiphilum*- of the Burkholderiales family of the Betaproteobacteria [58].

### 3.2. Phylogenetic Analysis Indicates More HGT for MaDH than the NMG Pathway

**Figure 3:**
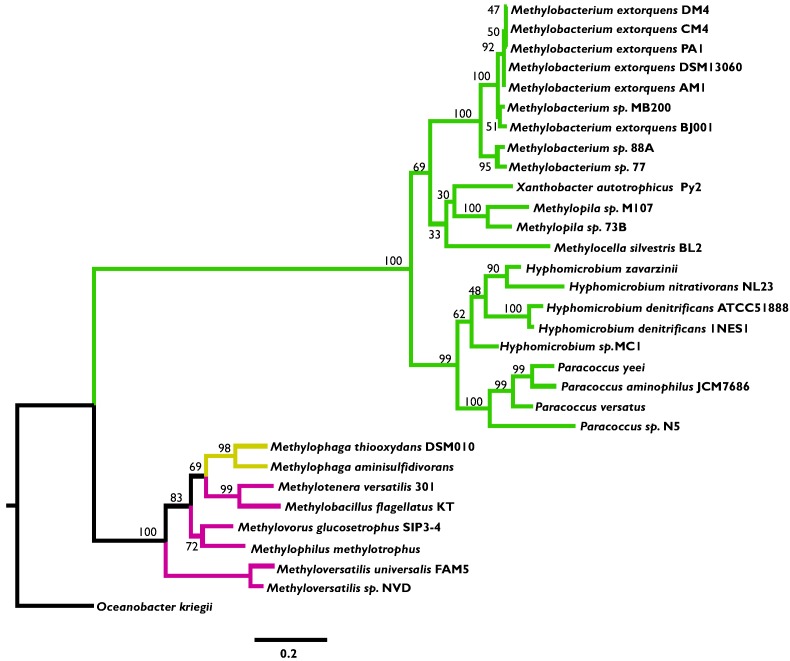
A maximum likelihood phylogeny of the *gmaS* amino acid sequence (encoding the first enzyme of the *N-*methylglutamate pathway) from sequenced methylotrophs of the Alpha—(green), Beta—(pink), and Gamma—(yellow) Proteobacteria using the *gmaS* sequence from *Oceanobacter kriegii* as the outgroup. Numbers adjoining nodes represent bootstrap support.

In order to determine whether the patchy distribution of MaDH across sequenced methylotrophic Proteobacteria indicates transfer by frequent HGT, we constructed maximum-likelihood (ML) amino acid phylogenies for key genes of each MA oxidation module. For the NMG pathway, we used *gmaS*, which encodes γ-glutamylmethylamide synthetase, the enzyme that catalyzes the first step of this MA oxidation pathway. The *gmaS* phylogeny (Figure 3) was mostly indicative of vertical inheritance, especially in strains belonging to the same species, but some notable instances of HGT were observed at higher levels of classification. For instance, *gmaS* sequences from strains of the *Hyphomicrobium* genera were found in a well-supported clade with strains of the *Paracoccus* genera rather than with other members of the Rhizobiales family.

**Figure 4:**
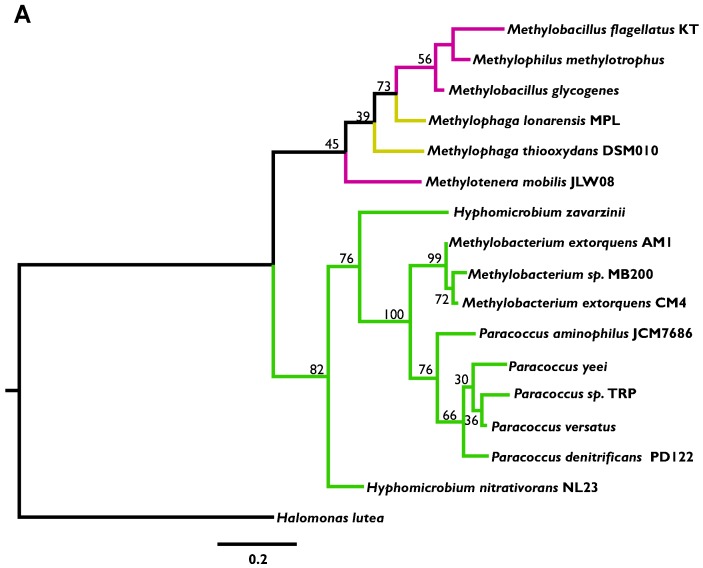

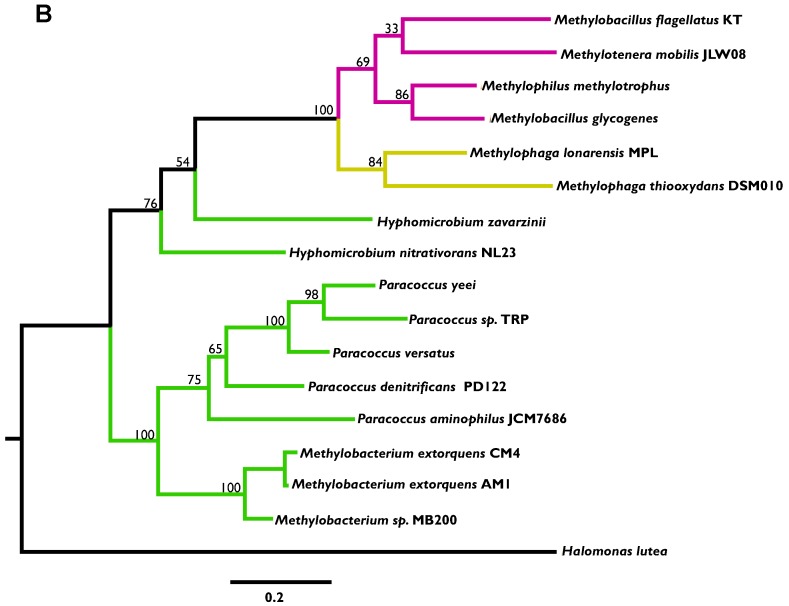
(**A**) A maximum likelihood phylogeny of the *mauB* amino acid sequence (encoding the large subunit of methylamine dehydrogenase) from sequenced methylotrophs of the Alpha—(green), Beta—(pink), and Gamma—(yellow) Proteobacteria using the *mauB* sequence from *Halomonas lutea* as an outgroup. (**B**) A maximum likelihood phylogeny of the *mauA* amino acid sequence (encoding the small subunit of methylamine dehydrogenase) from sequenced methylotrophs of the Alpha—(green), Beta—(pink), and Gamma—(yellow) Proteobacteria using the *mauA* sequence from *Halomonas lutea* as an outgroup. Numbers adjoining nodes represent bootstrap support.

For MaDH, we examined the ML amino acid phylogenies of genes encoding the large (*mauB)* and small (*mauA)* subunit of MaDH [14]. Although we had anticipated concatenating these genes, we found that each subunit has a distinct phylogenetic history, both of which show discordance not just within closely related strains but among different classes of the Proteobacteria as well (Figure 4A, 4B). Overall, analyses indicated that although neither pathway has experienced a fully vertical mode of inheritance, the degree of HGT appears to be greater for MaDH.

### 3.3. Highly Distinct Modes of Inheritance within Methylobacterium for Two Methylamine Oxidation Pathways

As the clade with the largest number of sequenced genomes, and also the most frequent occurrence of the NMG pathway, the *Methylobacterium* strains were examined in greater detail. In terms of the NMG pathway, phylogenetic analysis did not indicate any clear evidence of HGT when compared to the 16S rRNA phylogeny of *Methylobacterium* strains (Figure 3, Supplementary Figure S2). Additionally, the chromosomal location of this pathway in each strain was nearly identical (Supplementary Table S1). In contrast, the *mau* gene cluster was in three distinct chromosomal locations in each of the three *Methylobacterium* strains that express MaDH (Supplementary Table S2). These data strongly suggest that the *mau* gene cluster was introduced into this genus through three independent HGT events. Similarly, the *mauA* and *mauB* (Figure 4A,B) amino acid phylogenies were largely discordant with the16S rRNA phylogeny for *Paracoccus* strains (Supplementary Figure S3), whereas the *gmaS* amino acid phylogeny corroborated the notion of vertical inheritance (Figure 3).

### 3.4. The Methylamine Fitness of M. extorquens Species Is Dependent on the Metabolic Module Used for Methylamine Oxidation

**Figure 5:**
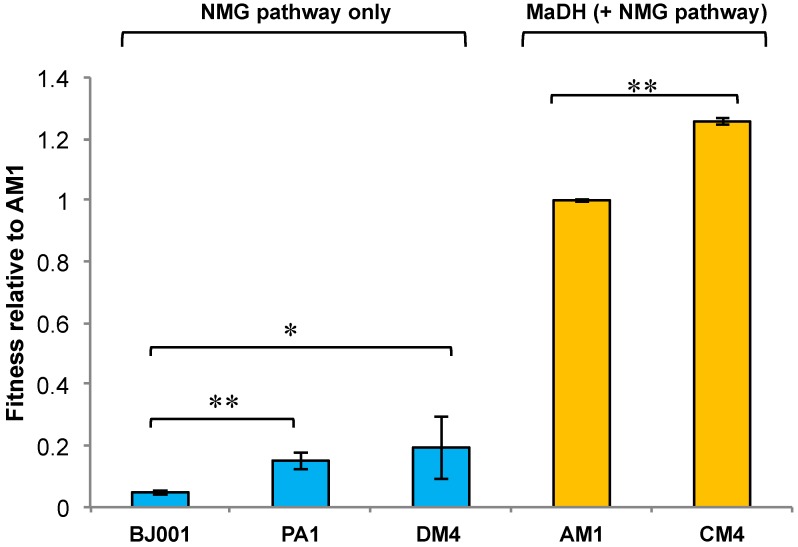
Methylamine fitness of *M. extorquens* species relative to AM1*.* Strains that use the *N-*methylglutamate pathway are depicted in blue and strains that use methylamine dehydrogenase (but also contain the *N-*methylglutamate pathway) are depicted in orange. Error bars depict the 95% confidence interval (C.I.) of the mean relative fitness determined by three replicate competition assays. ** *p* < 0.01 and * *p* < 0.05 for a significant difference in fitness on methylamine.

Although all strains of *M. extorquens* possess the NMG pathway, they differ in whether or not they possess MaDH (Figure 5) and we tested whether this had a consistent effect upon growth on MA. Competition assays relative to a PA1 strain expressing the fluorescent protein mCherry at a neutral locus [49] were used to measure the MA fitness of five different *M. extorquens* strains and subsequently determine whether it correlated with the presence of MaDH.

The resulting fitness values indicated a simple pattern: the two strains encoding MaDH, AM1 and CM4, were 5 to 20-fold more fit than PA1, DM4, and BJ001, all of which only possess the NMG pathway (Figure 5). Thus, although there were small but significant differences between strains with the same pathway for MA utilization, the dominant factor influencing fitness on MA was whether they possessed the horizontally acquired MaDH.

### 3.5. Experimental HGT of the mau Gene Cluster in M. extorquens PA1 Leads to an Instantaneous Increase in Fitness and Growth Rate on Methylamine

**Figure 6:**
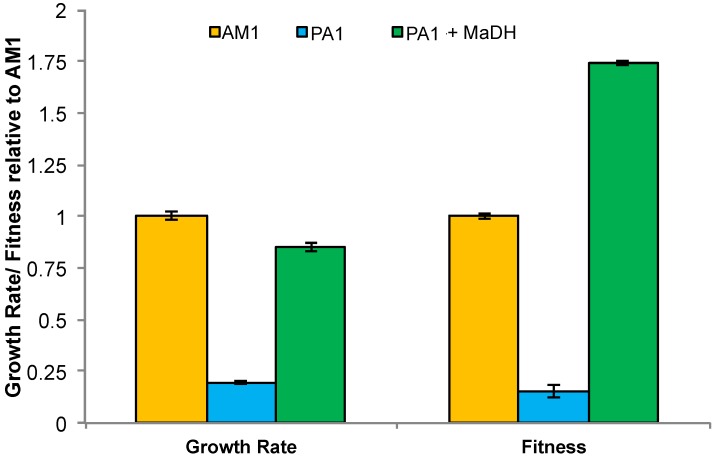
Methylamine growth rate and fitness of AM1 (light brown), PA1 (dark gray), and pAYC139^+^ PA1 expressing methylamine dehydrogenase (light gray) relative to AM1 (for growth rate) or mCherry AM1 (for fitness). Error bars each depict the 95% C.I. from three biological replicates.

Based on the evidence that there might have been three distinct HGT events of the *mau* gene cluster within the *Methylobacterium* clade, we sought to test if rapid growth on MA required substantial evolutionary refinement subsequent to HGT, or whether simply expressing MaDH could confer rapid growth. We simulated the HGT events that are likely to have occurred naturally for AM1 and CM4 by transforming a low-copy plasmid (pAYC139) [14] containing the *mau* gene cluster, downstream of the *Escherichia coli lac* promoter (P*_lac_*), from AM1 into PA1. The MA growth rate of the pAYC139^+^ transconjugant (in the absence of any antibiotic selection) was 4.3-fold greater than PA1 (*p* < 0.01) and only 15% lower than AM1 (*p* < 0.01) (Figure 6, Supplementary Table S3). Despite slightly slower growth than AM1, the pAYC139^+^ transconjugant was 75% more fit in competition assays relative to AM1 (*p* < 0.01) (Figure 6).

### 3.6. Laboratory-Based Evolution Did Not Lead to a Rapid Increase in Methylamine Fitness or Growth Rate for M. extorquens PA1

**Figure 7:**
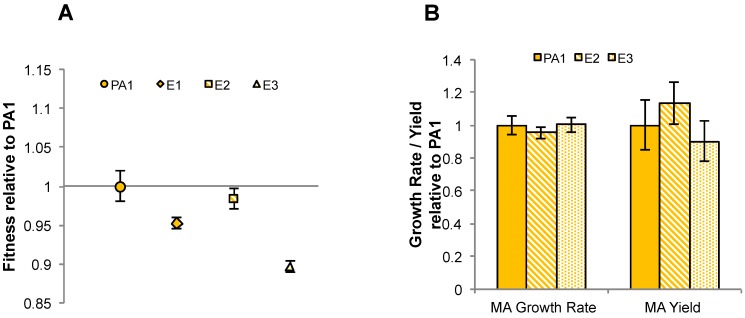
(**A**) MA fitness of Δ*cel M. extorquens* PA1 (orange) and an evolved isolate from each population (E1: diamond, E2: square and E3: triangle) relative to mCherry labelled strains of Δ*cel M. extorquens* PA1; (**B**) MA growth rate and yield (maximum OD_600_) of E2 (orange stripes) and E3 (orange dots) relative to PA1 (solid orange).

Although introduction of MaDH instantly enabled fast growth of PA1 on MA, this did not indicate whether or not the NMG pathway could also rapidly evolve to improve MA growth in PA1 if selection were to reward doing so. To mimic this process in the laboratory, we initiated serial transfers of PA1 in media with MA as the sole carbon and energy source for 150 generations. Three replicate populations of PA1 were grown in minimal media with 20 mM MA with transfers every 3.5 days. Serial transfer regimes select primarily for mutants with enhanced growth rates [39,59]. Single isolates from each evolved population were obtained, and have been denoted as E1, E2, and E3, and fitness on MA was measured relative to the ancestor expressing the fluorescent protein mCherry (Figure 7A).

In sharp contrast to previous instances of experimental evolution with poor-growing *M. extorquens* strains where growth improved within 100 generations [39,40,51], none of the evolved isolates were more fit than the ancestor and, to our surprise, E1 and E3 actually had significantly lower competitive fitness on MA (*p* = 0.01 and *p* < 0.01, respectively). To check whether the strains isolated were anomalous in their performance, we also performed the same competition with each of the evolved populations (denoted as P1, P2, and P3) against the labeled ancestor. Two of the three populations were significantly less fit than the ancestor (P1 and P3; *p* < 0.01 in each case) and the fitness of one population was not significantly different from the ancestor (P2; *p* = 0.17) (Supplementary Figure S4). We also examined the growth rates and yield (assayed via max OD_600_) of two of the evolved isolates (Figure 7B, Supplementary Table S3) and neither of these traits changed significantly either (*p* > 0.2 for all tests). Altogether, these data indicate that fitness or growth rate of PA1 on MA did not increase despite 150 generations of selection for fast growth.

## 4. Discussion

In this study, we uncovered the evolutionary history and the apparent adaptive constraints underlying the current distribution of MaDH and the NMG pathway, two functionally degenerate routes for MA oxidation, in methylotrophs. First, a systematic survey of these two pathways in sequenced methylotrophs belonging to the Proteobacteria indicated that the NMG pathway is more abundant and universally distributed across methylotrophic Proteobacteria, whereas the *mau* gene cluster that encodes MaDH and ancillary proteins [14] is observed in fewer strains and has a patchy distribution characteristic of transmission primarily by HGT (Figure 2). Even though the amino acid phylogeny of *gmaS*, encoding one of the three enzymes (GMAS) of the NMG pathway was not completely congruent with vertical descent, the amino acid phylogenies of the genes encoding the large (*mauB*) and small subunit (*mauA*) (Figure 3, Supplementary Figure S3) of MaDH indicated much more rampant HGT within and across different clades of the Proteobacteria. A marked distinction in the mode of transmission for these two MA oxidation pathways was evident within bacterial strains belonging to single genera. For strains within the *Paracoccus* (Supplementary Figure S3) and *Methylobacterium* (Supplementary Figure S2) clades, the *gmaS* phylogeny was congruent with the 16S rRNA phylogeny (Figure 3), but the *mauA* and *mauB* phylogenies were very discordant (Figure 4A,B). Furthermore, a complete lack of chromosomal synteny around the *mau* gene cluster (Supplementary Table S2) between three closely related *Methylobacterium* strains was consistent with three unique HGT events into this single clade.

Based on the observation that the distribution of these MA oxidation pathways can vary significantly across closely related strains, we tested whether this genotypic variation has a phenotypic influence on MA growth in well-characterized *M. extorquens* strains (Figure 2). The MA fitness of *M. extorquens* strains was quite variable: Strains like AM1 and CM4 that also encode MaDH had at least five fold or greater fitness on MA compared to strains like PA1, BJ001, and DM4 that only encode the NMG pathway (Figure 5). These phenotypic data strongly suggest that independent of genomic context, each of these two MA oxidation pathways has a dramatic influence on the MA growth rate of a bacterium.

By mimicking HGT and introducing the *mau* gene cluster [14] encoding MaDH on a plasmid in PA1, we found that the MA fitness and growth rate of PA1 increased instantaneously and the resulting strain had higher fitness than AM1 (Figure 6). Reduced lag time and/or an increased survival in stationary phase are likely to have contributed to higher fitness of the pAYC139^+^ PA1 transconjugant despite slower MA growth rates when compared to AM1. Additionally, a non-native promoter enhancing the expression of the *mau* gene cluster on an extra chromosomal element may have influenced fitness as well. Overall, combined with finding multiple, independent acquisitions of the *mau* cluster in sequenced *M. extorquens* strains, these results suggest that it is very easy to incorporate MaDH-dependent use of MA without the need for substantial, subsequent evolutionary refinement. The facile transfer of MaDH also corroborates the broader concept of methylotrophy as being modular in terms of both physiology and evolution [12,13]. This outcome contrasts with previous studies in which two different metabolic modules were shown to require significant evolutionary refinement after HGT for optimal functional expression [39,40,49] in members of the *M. extorquens* species. In one example, replacement of the H_4_MPT-dependent formaldehyde oxidation pathway in AM1 with a non-orthologous route using a series of glutathione-dependent reactions decreased the growth rate on methanol three-fold [39,50] and recently it was also shown that transferring the dichloromethane oxidation module (*dcmA*) from *M. extorquens* DM4 only permitted modest to no growth on dichloromethane in other *M. extorquens* strains [49].

Given that introduction of MaDH led to rapid growth of PA1 on MA, we were surprised by how modest the selective response for improved use of the endogenous NMG pathway was. Most laboratory evolution experiments [39,40,42,51,60], including those with other *M. extorquens* strains [40,51,60], have shown that there tends to be rapid evolutionary adaptation when either the environment or the genotype slow down growth rates. Despite poor growth on MA initially, when replicate populations of PA1 were evolved on MA for over 150 generations, the competitive fitness and growth rates of the evolved isolates or populations did not improve. In contrast, a 40% improvement in growth rate was observed within just 72 generations of experimental evolution when the aforementioned engineered strain, in which the H_4_MPT-dependent pathway was replaced with a glutathione-dependent pathway, was evolved on methanol [39,50]. It is worth pointing out that even though no significant growth improvements were observed after 150 generations of evolution of PA1 on MA, it does not preclude the possibility that, over extended periods of time, one would not observe adaptive mutations leading to large jumps in fitness, as has been occasionally observed in other systems [59,61]. Regardless of the underlying physiology, this example of poor initial growth and low evolvability of the NMG pathway argues against the universality of the general evolutionary heuristic [41,42] that fitness gain for any given strain is negatively correlated with its initial fitness and primarily independent of the starting genotype.

Given the apparent ease of achieving fast MA growth upon acquiring MaDH, it is perhaps surprising that this metabolic module is less abundant in comparison to the NMG pathway in our analysis of sequenced genomes as well as metagenomic datasets from various natural ecosystems [4,7,35,36]. An ecological basis for the abundance of the NMG pathway, despite being the suboptimal route for MA utilization under laboratory growth conditions, remains to be understood. Furthermore, it is unclear what selective forces allow so many methylotrophs to maintain both of these degenerate MA utilization routes (Figure 2) in their genome. In conclusion, MaDH appears to be yet another excellent example of how HGT can play a major role in the evolutionary dynamics of microorganisms in nature by rapidly opening the door to new physiological possibilities that can overcome adaptive constraints posed by existing systems.

## Supplementary Files

Supplementary File 1
