# Supplementary material for: Experimental Horizontal Gene Transfer of Methylamine Dehydrogenase Mimics Prevalent Exchange in Nature and Overcomes the Methylamine Growth Constraints Posed by the Sub-Optimal N-Methylglutamate Pathway
Source: Microorganisms. 2015 Mar 10;3(1):60–79. doi: 10.3390/microorganisms3010060 (PMC5023228; doi:10.3390/microorganisms3010060)
Supplement: Supplementary File 1 [file microorganisms-03-00060-s001.docx]

**Supplementary Information**


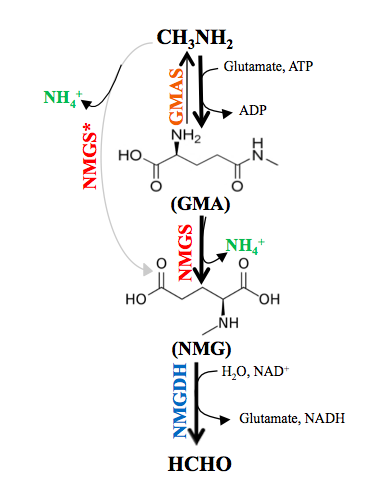


**Figure S1.** The topology of the *N-*methylglutamate pathway in *M. extroquens* species has been shown to be semi- linear; *N-*methylglutamate synthase is capable of synthesizing *N-*methylglutamate from γ-glutamylmethylamide (black arrow) as well as methylamine (gray arrow) albeit only in the Δ*gmas* mutant.


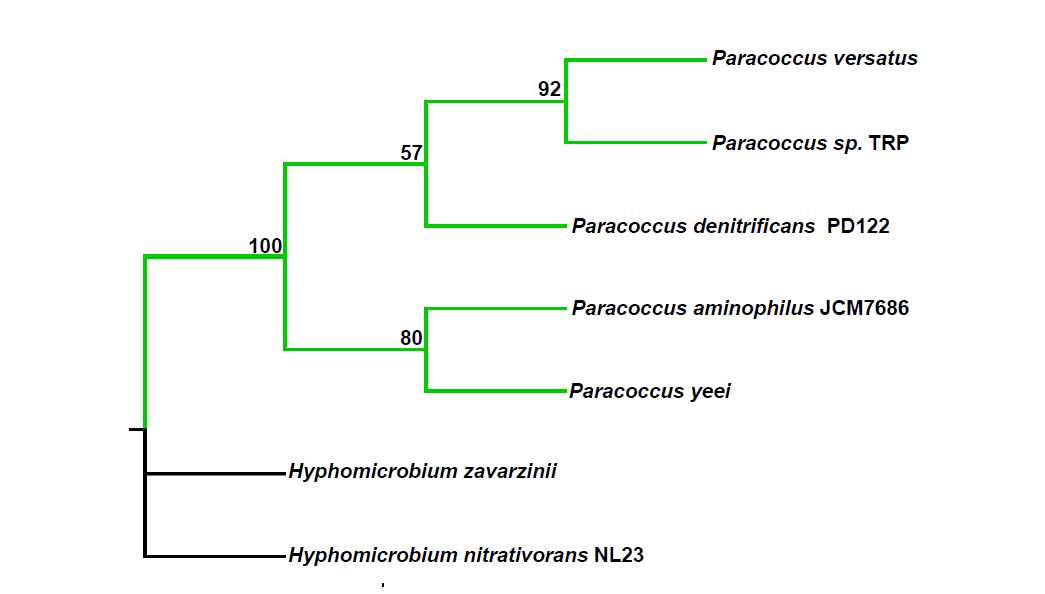


**Figure S2.** A ML phylogeny of the nucleotide sequence of 16S ribosomal RNA sequences of *Paracoccus* strains. 16S ribosomal RNA sequences of *Hyphomicrobium* strains was used as an outgroup and the node labels represent the bootstrap support. The branch lengths have been transformed to depict the relationship between closely related strains.


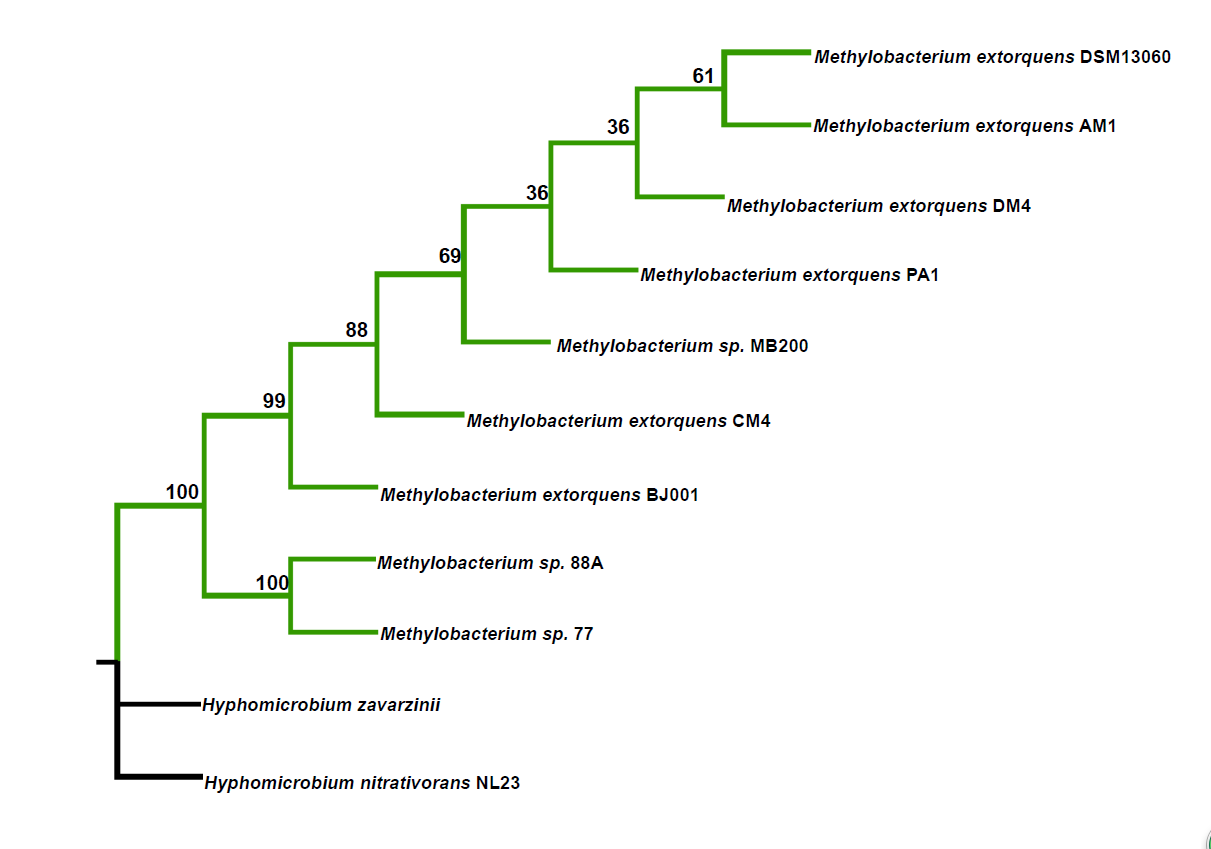


**Figure S3.** A ML phylogeny of the nucleotide sequence of 16S ribosomal RNA sequences of *Methylobacterium* strains. 16S ribosomal RNA sequences of *Hyphomicrobium* strains was used as an outgroup and the node labels represent the bootstrap support. The branch lengths have been transformed to depict the relationship between closely related strains.


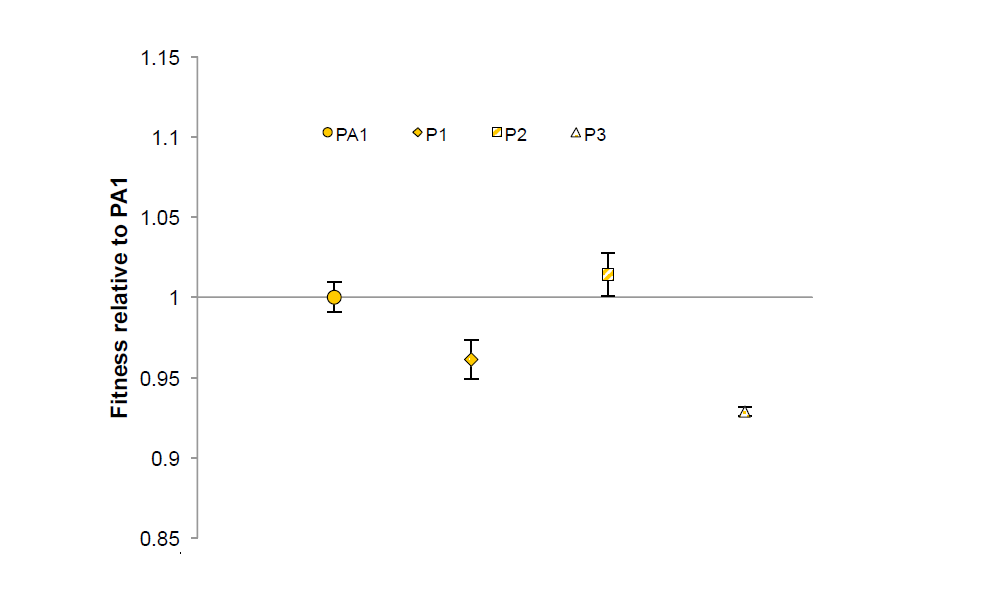


**Figure S4.** Competitive fitness of the three methylamine evolved populations (denoted as P1, P2 and P3) of *M. extorquens* PA1 relative to the ancestral strain expressing the fluorescent protein mCherry in media with 20 mM methylamine as the sole carbon and energy source.

**Table S1.** Chromosomal location and genes flanking the NMG pathway in strains of Methylobacterium species.

| **Strain** | **Location of the NMG Pathway Genes** | **Flanking Genes** |
| --- | --- | --- |
| *M. extorquens* PA1 | Mext_1651 to Mext_1659 | Mext_1649 (putative sensor kinase with PAS/PAC and response receiver regulator receiver domains) and Mext_1662 (putative heme peroxidase with hemolysin type calcium binding domain) |
| *M. extorquens* AM1 | Meta1_1545 to Meta1_1553 | Mext_1543 (putative sensor kinase with PAS/PAC and response receiver regulator receiver domains) and Mext_1555(putative phosphoesterase or phosphohydrolase) |
| *M. extorquens* CM4 | Mchl_1933 to Mchl_1941 | Mchl_1931(putative sensor kinase with PAS/PAC and response receiver regulator receiver domains) and Mchl_1943 (conserved protein of unknown function) |
| *M. extorquens* DM4 | METDI_2319 to METDI_2327 | METDI_2317(putative sensor kinase with PAS/PAC and response receiver regulator receiver domains) and METDI_2330 (putative heme peroxidase with hemolysin type calcium binding domain) |
| *M. extorquens* DSM13060 | AGJKv1_5633 to AGJKv1_5639  (*mgdA*, *mgdB*, all *mgs* genes and *gmaS*)  AGJKv1_2666 to AGJKv1_2667 (*mgdD* and *mgdC*) | AGJKv1_5631 (SMC domain protein) and AGJKv1_5641 (translation initiation factor IF-2) AGJKv1_2665 ((putative sensor kinase with PAS/PAC and response receiver regulator receiver domains) |
| *M. extorquens* BJ001 | MEXB_1770 to MEXB_1777 | MEXB_1779 (putative sensor kinase with PAS/PAC and response receiver regulator receiver domains) and MEXB_1760 (putative sensory box/GGDEF family protein) |

**Table S2.** Chromosomal location and genes flanking the mau gene cluster in strains of *Methylobacterium* species

| **Strain** | **Location of *mau* Genes** | **Strand** | **Flanking Genes** |
| --- | --- | --- | --- |
| *M. extorquens* AM1 | Meta1_2769 to Meta1_2779 | +1 | Meta1_2765 (small multi-drug efflux pump, DMT superfamily) and Meta1_2784 (protein of unknown function) |
| *M. extorquens* CM4 | Mchl_0556 to Mchl_0566 | -1 | Mchl_0551 (putative transcriptional regulator, XRE family) and Mchl_571 (TonB-dependent receptor) |
| *Methylobacterium.* sp. MB200 | AMXUv1_780086 to AMXUv1_780096 | +1 | AMXUv1_780085 (aminomethyltransferase) and AMXUv1_780097 (conserved exported protein of unknown function) |

**Table S3.** Growth rate of strains in hypho minimal media with 20 mM methylamine as the sole carbon and energy source.

| **Strain** | **Growth Rate on Methylamine ± Standard Error of three Biological Replicates (h^-1^)** |
| --- | --- |
| *M. extorquens* AM1 (CM2720) | 0.207 ± 0.001 |
| *M. extorquens* PA1 (CM2730) | 0.041 ± 0.006 |
| *M. extorquens* PA1 + pAYC139 | 0.176 ± 0.001 |
| Evolved Isolate from Population 2 (E2) | 0.039 ± 0.001 |
| Evolved Isolate from Population 3 (E3) | 0.041 ± 0.003 |
